# Mechanochemical Preparation of Superabsorbent Materials from Okara and Itaconic Acid

**DOI:** 10.3390/molecules31111830

**Published:** 2026-05-26

**Authors:** Abdul Hafeez, Gyanendra Sharma, Romain Milotskyi, Hao Wang, Akihiro Shinku, Naoki Wada, Kenji Takahashi

**Affiliations:** 1 Division of Biological Science and Technology, Graduate School of Natural Science and Technology, Kanazawa University, Kakuma-Machi, Kanazawa 920-1192, Ishikawa, Japan; h-wang@se.kanazawa-u.ac.jp (H.W.); shinaki0925sitwa@stu.kanazawa-u.ac.jp (A.S.); 2 Faculty of Biological Science and Technology, Institute of Science and Engineering, Kanazawa University, Kakuma-Machi, Kanazawa 920-1192, Ishikawa, Japan; sharmag-19@se.kanazawa-u.ac.jp (G.S.); romain-mi@se.kanazawa-u.ac.jp (R.M.); naoki-wada@se.kanazawa-u.ac.jp (N.W.)

**Keywords:** soybean waste, Okara, itaconic acid, superabsorbent polymers, bio-based, mechanochemistry, reactive extrusion

## Abstract

In this study, a green technique of mechanochemistry was used to prepare superabsorbent polymers (SAPs) from soybean waste (Okara) and bio-based bifunctional itaconic acid (ItA) in a solventless melt-reactive esterification reaction using reactive extruder. SAPs were produced by reaction of ItA with Okara at 120 °C with and/or without the use of crosslinker *N*,*N′*-methylenebis(acrylamide) (MBA) in the presence or absence of free radical initiator, potassium peroxodisulfate (KPS). By varying the amounts of ItA and Okara, the effect of MBA and KPS was investigated on water absorption. The esterification of Okara with ItA was confirmed by attenuated total reflectance–Fourier-transform infrared (ATR-FTIR) spectroscopic measurements, while the structural characterization was done using X-ray diffraction, thermal gravimetric analysis, and scanning electron microscopy. Among the twelve SAPs formulations, the highest water absorption of 35.6 g/g of SAP was shown by SAP prepared from Okara/ItA in a ratio of 1 g/3.5 g and crosslinked with 0.5 wt% MBA. All the SAPs showed moderate centrifuge water retention (CWR) capabilities which show their potential for application in sustainable agriculture.

## 1. Introduction

The most successful commercial SAPs such as poly(acrylate), polyacrylonitrile, polyacrylamide, and poly(methacrylate) are derived from petroleum-based monomers. The robust nature of C-C bonds in SAPs’ molecular chains made them practically non-biodegradable and poorly biocompatible, resulting in an environmental crisis [1,2]. At present the global efforts towards carbon neutrality necessitate a reduction in reliance on petrochemicals and the pursual of alternative bio-based materials. The shift from petroleum-based SAPs to bio-based SAPs is witnessing a different level of success and challenges [3,4]. Researchers are investigating SAPs prepared from natural polymers such as polysaccharides, cellulose, starch, chitosan, carrageenan, alginate, and gum arabic [5,6,7]. SAPs are widely used in hygiene products, agriculture, biomedical materials, wastewater treatment, food packaging, and controlled drug delivery due to their exceptional water absorption and retention capacities [8,9,10]. It has been found that the SAPs prepared from natural polymers show better swelling capacity only when grafted with petroleum-based monomers such as acrylic acid and acrylamide. Although numerous studies and endeavours have been done to prepare bio-based SAPs, suitable replacement of acrylic acid-based SAPs has not been identified yet. In this context, itaconic acid (ItA) or methylene succinic acid or (2-methylene butane-1,4-dicarboxylic acid) a bio-renewable bifunctional dicarboxylic acid, can be a surrogate of acrylic acid. In fact, in 2004 the U.S. Department of Energy listed it as one of the top value-added chemicals from biomass [11,12]. It is being produced industrially by fermentation of polysaccharides with *Aspergillus terreus* with a price less than 2 US$ per kg [13,14]. The presence of two carboxylic functionalities and an α,β-unsaturated double bond makes it a promising candidate for preparation of SAPs [15,16]. However, the homo-polymerization of ItA proceeds sluggishly due to combined effects of steric and electronic structures, [17,18] and therefore, yield only about 32% conversion, [19] which has hindered its widespread adoption as an alternative to acrylic acid.

Okara is a soybean residue after making tofu or soymilk. The worldwide production of Okara is approximately 14 million tons per annum [20,21,22]. Every kg of Tofu produces 1.2 kg Okara [23]. It has anti-nutrient effects and low sensory acceptance; therefore, only a small fraction of Okara is used as animal feed or in other applications, and most of it is either dumped or discarded [22,24,25]. Conversion of this biowaste to SAPs would have a dual effect of solving the problem of waste and retaining the water for agricultural plants [26,27].

On the other hand, mechanochemistry is a green technology which is used to do reactions in solventless systems. It utilizes mechanical forces like compression, shear, and compaction to induce chemical reactions, thereby reducing the necessity for auxiliary solvents [28]. It has been listed by IUPAC as one of the ten innovations that can shape the future world [29,30]. Therefore, the aim of this study was to synthesize and characterize fully bio-based SAPs using green technique of mechanochemistry in a solventless melt-reactive esterification process by grafting ItA onto the soybean industry waste product, Okara. An attempt has been made to find an alternative to acrylic acid-based SAPs by minimizing the solvents and valorization of Okara.

## 2. Results and Discussion

The mechanochemical reaction was confirmed by ATR-FTIR spectroscopy and structural characterization was performed with X-ray diffraction analysis and scanning electron microscopy (SEM). The appearance of absorption band of C=O ester bond in ATR-FTIR spectra along with disappearance of peak of vinyl =CH_2_ group of ItA indicated that the esterification occurred successfully [27]. The surface morphology by SEM also revealed that by changing the reaction conditions, the morphology also changed. The porous surface shown in SEM justified the water absorption due to capillary action. The reaction in the presence of MBA crosslinker benefited water absorption only when a higher amount of ItA was used. In case of reactions in which both KPS and crosslinker MBA were used, water absorption was reduced. An interesting correlation was found between the mass yield, Y_Ok_ (%), and water absorption. Those SAP samples which gave lower yield showed higher water absorption, indicating that the larger molecules with grafted ItA were better water absorbers. The highest swelling capacity of 35.6 g/g was shown by SAP (Entry 3) which was comparable to the centrifuge retention capacity of SAP prepared by copolymerization of ItA with acrylic acid SAPs [31].

### 2.1. Mechanochemical Synthesis and Structural Characterization

**Figure 1:**
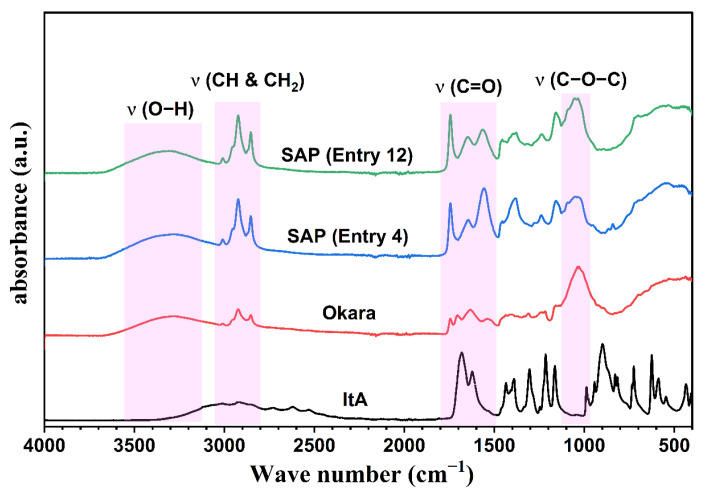
Normalized ATR mode FTIR spectra of raw materials (ItA and Okara) and their SAPs (Entries 4 and 12).

For mechanochemical reaction 120 °C was used because below this temperature kneading in the microcompounder was difficult due to high torque, which caused the kneading screw to stop. At this temperature ItA melted under shearing and compressive forces and the reaction proceeded smoothly. Since ItA has two –COOH groups, esterification of one –COOH group leaves another carboxylic group free. The conversion of –COOH acid functionalities to carboxylate salts –COO^-^ make them hydrophilic groups, which are beneficial for water absorption. After the reaction, the structural and chemical changes were identified using ATR-FTIR spectra. SAP samples with different amounts of crosslinking agent, MBA, and free radical initiator, KPS, were synthesized. Figure 1 shows the spectra of Okara, its esterification reagent, and SAPs prepared with the use of KPS radical initiator and MBA crosslinker. SAP (Entry 4) was prepared with the use of 0.5 wt% KPS and 0.5 wt% MBA crosslinker, while SAP (Entry 12) was prepared with the use of 1.0 wt% KPS and 0.5 wt% MBA crosslinker. The ATR-FTIR spectra of remaining SAPs can be found in ESI (Figures S1 and S2).

The spectrum of ItA showed characteristic absorption peaks at 1679 cm^−1^ (C=O), 1619 cm^−1^ (C=C stretching) and 898 cm^−1^ (vinyl =CH_2_ bending). Okara, which was a mixture of polysaccharides, proteins, lipids and other minor components, showed a broad absorption peak at 3257 cm^−1^ due to O-H stretching of polysaccharides, and 2917–2850 cm^−1^ due to C-H stretching of aliphatic CH_2_ and residual lipids [27]. The presence of absorption peak at 1716 cm^−1^ was ascribed to free acidic groups as well as esterified pectin, while the peaks at 1698 cm^−1^ and 1625 cm^−1^ were associated with amide I and amide II linkages [27]. The medium broad peak at 1527 cm^−1^ was due to N-H and C-N bending and stretching [32]. The intense peak in the fingerprint region at 1026 cm^−1^ was due to the cellulose-rich structure of Okara [26]. After mechanochemical esterification of Okara and ItA, a significant change in spectra was observed. The absorption peak at 1679 cm^−1^ of C=O in ItA disappeared and a new peak appeared at 1741 cm^−1^ with significant intensity which indicated a successful esterification reaction. The absence of peaks at 1619 cm^−1^ and particularly 898 cm^−1^ due to vinyl =CH_2_ peak indicated successful polymerization [33]. The peaks at 1633 cm^−1^ and 1548 cm^−1^ arose due to asymmetric and symmetric stretching vibrations of carboxylate (-COO^−^), respectively. Similarly, stronger CH_2_ stretching vibrations at 2919–2852 cm^−1^ provided evidence for the formation of a saturated polymeric backbone [33]. During the neutralization process and subsequent washing, it was assumed that the lipids, pectin and other minor components were removed. The presence of a fingerprint absorption peak at 1029 cm^−1^ in the final product along with a broad O-H stretching vibrational peak at 3300 cm^−1^ provided strong evidence that the ItA was grafted onto the cellulosic backbone [34].

**Figure 2:**
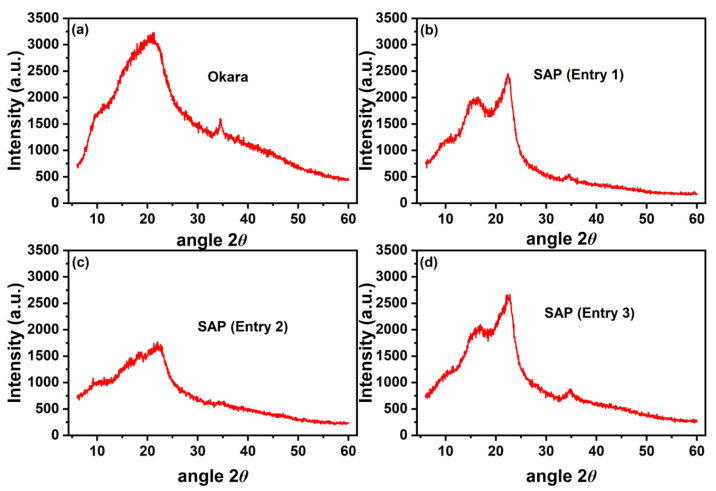
X-ray diffractograms of Okara and its SAPs (**a**) Okara, (**b**) SAP (Entry 1) prepared without KPS and MBA crosslinker, (**c**) SAP (Entry 2) prepared with KPS, and (**d**) SAP (Entry 3) prepared with MBA crosslinker.

The X-ray diffraction patterns of Okara and its different mechanochemical products with ItA give useful information about the structural properties of the prepared SAPs. Although Okara consists of cellulose, protein and other components, the crystallinity mainly comes from cellulose. Mechanochemical reactions can increase or decrease the crystallinity depending on whether they strengthen ordered packing or disrupt cellulose packing [28,35,36]. Figure 2 shows the X-ray diffractograms of Okara, SAPs (Entries 1, 2, and 3). It was interesting to note that when Okara and ItA were reacted in a 1:3.50 weight ratio in absence of synthetic crosslinker and free radical initiator, their product, SAP (Entry 1), had more crystallinity than Okara. When they were reacted in the presence of 0.5 wt% KPS initiator, SAP (Entry 2) had less crystallinity as compared to SAP (Entry 1). On the other hand, when they were reacted in the presence of 0.5 wt% MBA crosslinker, their SAP (Entry 3) showed more crystallinity than SAP (Entry 2). In a SAP (Entry 4) (Figure S3), prepared in the presence of both 0.5 wt% KPS and 0.5 wt% MBA, the product showed least crystallinity. A similar trend was seen in all other SAPs which suggested that when Okara reacted with ItA, in absence of KPS and MBA, the ester linkages were formed between hydroxyl groups of polysaccharides accompanied by removal of amorphous regions, thereby relative fraction of crystalline regions was increased, even though the absolute crystallinity remain unchanged.

When Okara was reacted with ItA in the presence of KPS free radical initiator, it was observed that the crystallinity of the prepared SAP decreased (Figure 2 and Figure S3). This suggested that the polysaccharides’ backbone chain scission happened due to free radicals’ attack on chains, [37] because broken chains lose their ability to align into crystalline regions, therefore, crystallinity reduced. In the presence of KPS, radical polymerization of ItA itself happened, forming poly(itaconic acid) [38]. At the same time, apart from hydrolysis of amorphous regions of polysaccharides due to ItA during reaction, grafting of poly(itaconic acid) onto Okara initiated by free radicals further reduced the crystallinity.

When Okara reacted with ItA in the presence of MBA crosslinker without KPS, the relative crystallinity increased again. This can be explained by the fact that the reaction of crosslinker with accessible amorphous regions of polysaccharide microfibrils created a constraint which restricted mobility of microfibrils, and thus reinforced crystalline micro-domains, thereby increasing the relative crystalline fraction in the SAPs [39].

The SAPs prepared from reactions of Okara with ItA in the presence of both KPS and MBA showed the least crystallinity. The combined effect of KPS and MBA had a very interesting influence on crystallinity. KPS initiated free radical polymerization of ItA to produce soft and amorphous poly(itaconic acid) chains which were grafted onto Okara and contributed to lowering the crystallinity. At the same time free radicals caused polysaccharides’ chain scission events. Although MBA tried to crosslink and form a network, broken chains reduced efficiency. The concomitant effect of MBA crosslink-induced ordering and KPS free-radical-induced disordering resulted in an overall reduction in crystallinity [40,41].

### 2.2. Thermal Analysis

**Figure 3:**
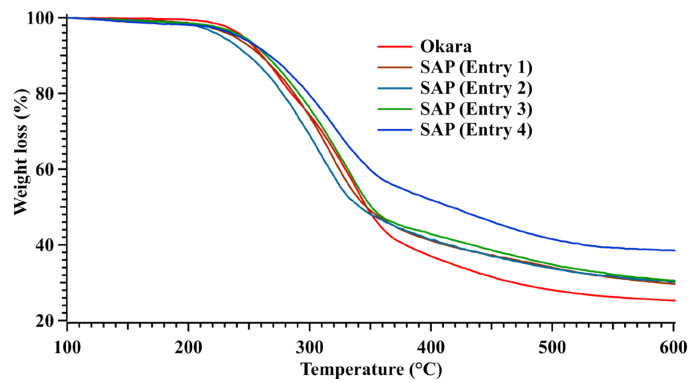
Thermogravimetric analysis (TGA) graphs of Okara and its SAPs synthesized with ItA with and/or without KPS and MBA.

TGA was used to evaluate the thermal behaviour of SAPs and Okara. The TGA curves of SAPs (Entries 1, 2, 3 and 4) are shown in Figure 3, and key parameters are summarized in Table 1. The temperature corresponding to 5% weight loss, often considered as the onset of thermal degradation (T_onset_), ranged from 228 °C to 249 °C. The TGA (Figure 3 and Figure S4) and derivative thermogravimetric (DTG) curves (Figure S5) revealed a maximum degradation rate (T_max_) at 314.19 °C to 317.25 °C for SAP (Entries 1, 5 and 9), corresponding to the breakdown of the polymeric backbone [16]. The shoulder peaks in DTG curves centred around 275 °C to 286 °C were associated with degradation of proteins in the SAPs. At 600 °C, the char residue content of SAPs varied between 28.99% and 39.30%, which was higher than char residue of Okara (25.27%). The samples SAP (Entry 8) and SAP (Entry 11) exhibited the highest thermal stability with 39.30% and 39.13% char residues, respectively (Figure S4). This indicated that they possessed higher inorganic content and the formation of stable carbonaceous structures [42].

### 2.3. Surface Morphology

**Figure 4:**
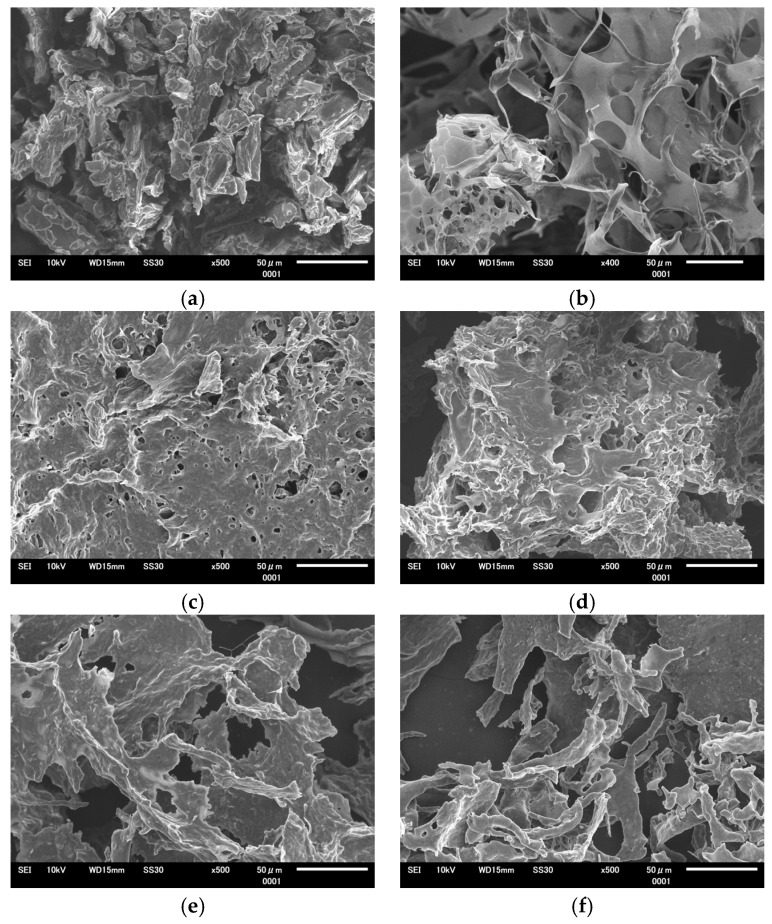
Scanning electron micrographs of (**a**) Okara, (**b**) SAP (Entry 3), (**c**) SAP (Entry 5), (**d**) SAP (Entry 8), (**e**) SAP (Entry 10), and (**f**) SAP (Entry 11).

Figure 4 shows the SEM images of unmodified Okara, and its SAPs prepared with ItA. Each micrograph showed a distinct change in morphology with changing the ratio of reactants, KPS initiator and MBA crosslinker. The SEM image of Okara showed that it possessed compact, dense and smooth flake-like texture with no visible voids [43]. After its reaction in the microcompounder with ItA, the best texture was shown by SAP (Entry 3), which was prepared with 0.5 wt% MBA crosslinker without the KPS radical initiator. The smooth, sponge-like, loosely interconnected pores were seen in the SAP (Entry 3) micrograph which justifies the highest water absorption shown by this sample, whereas the texture of SAP (Entry 5), which was prepared without KPS and MBA, changed to coarse and rough with smaller voids [44]. In the presence of 0.5 wt% MBA and 2 wt% KPS initiator in SAP (Entry 8), the broken flaky texture transformed into cohesive coarse dense flakes, which further reduced the water absorption [45]. However, in the presence of 1 wt% KPS initiator in SAP (Entry 10), its surface texture transformed to wrinkled and flaky. When the ratio of Okara was increased and ItA decreased in SAP (Entry 11) under same reaction conditions in the presence of 0.5 wt% MBA, the delicate loosely interconnected structure crumbled and turned to broken flakes, which showed why it absorbed less water.

### 2.4. Measurement of Water Absorption

**Figure 5:**
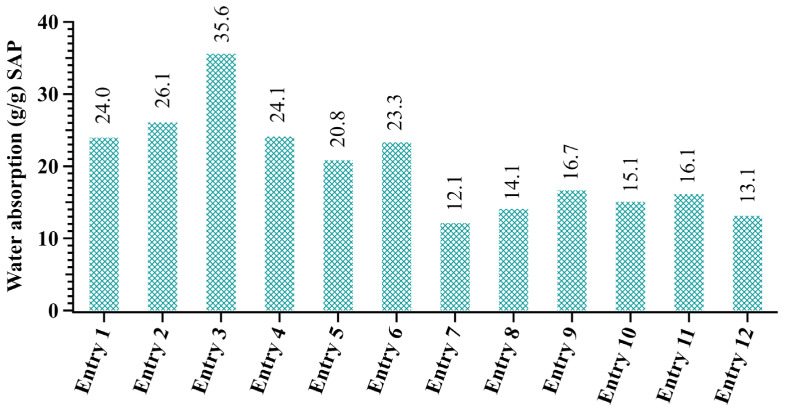
Free swelling capacity of SAPs synthesized by mechanochemical esterification of Okara and ItA.

The free swelling capacity of synthesized SAPs was evaluated in distilled water. As shown in Figure 5, the Okara-based SAPs showed a moderate swelling capacity, which reached a maximum of 35.6 g/g in distilled water. This indicated the successful incorporation of hydrophilic -COOH functionalities into the base polymeric network. The hydrophilic carboxylate groups incorporated by grafting of ItA onto the Okara, increased the hydrophilic character of the SAPs. The osmotic pressure gradient between the internal polymeric network and external water was the primary reason for free water absorption in SAP [46]. All the remaining samples exhibited free swelling absorption ranging between 12.1 g/g to 35.6 g/g. The higher the free swelling capacity of the SAP, the larger the amount of water that remained in the SAP structure after taking out the tea bag from the water. It was observed that the KPS and MBA had a different influence on the water absorption. For example, the SAPs prepared from reaction of 3.50 g of ItA (SAPs (Entry 1 to Entry 4)) and 3.37 g of ItA (SAPs (Entry 5 to Entry 8)) with 1.0 g and 1.40 Okara, respectively, showed increase in water absorption with the use of KPS initiator. However, when the amount of Okara was further increased and the amount of ItA was decreased then the reverse trend was observed. For example, the SAPs (Entry 9) and SAP (Entry 10), which were prepared using 1.78 g/2.86 g ratio of Okara/ItA exhibited lower water absorption with the use of KPS initiator. On the other hand, the use of MBA crosslinker, in general, reduced the water absorption except in the case of SAP (Entry 3). For example, the comparison of SAP (Entry 5) and SAP (Entry 7) showed that the water absorption reduced from 20.8 g/g to 12.1 g/g with the use of MBA crosslinker. Similarly, the water absorption of 16.7 g/g in SAP (Entry 9) reduced to 16.1 g/g SAP (Entry 11) with the use of MBA crosslinker. It is notable that the SAPs prepared with the use of both KPS and MBA were the lowest performers. In the presence of crosslinker, the reduced water absorption was probably due to the increased density of crosslinking, as also evidenced by SEM morphology. The SAP sample prepared from 1.0 g/3.50 g ratio of Okara/ItA in the presence of 0.5 wt% MBA showed the highest performance, indicating the optimal crosslinking density among all the samples [16]. This was supported by the SEM morphology.

**Figure 6:**
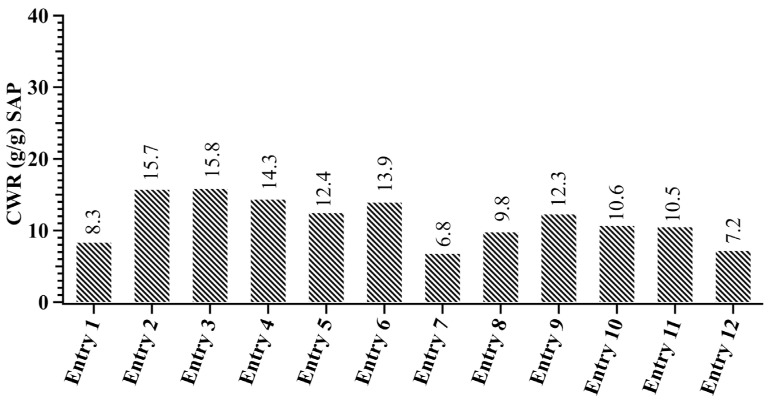
Centrifugation water retention of SAPs synthesized by mechanochemical esterification of Okara and ItA.

**Figure 7:**
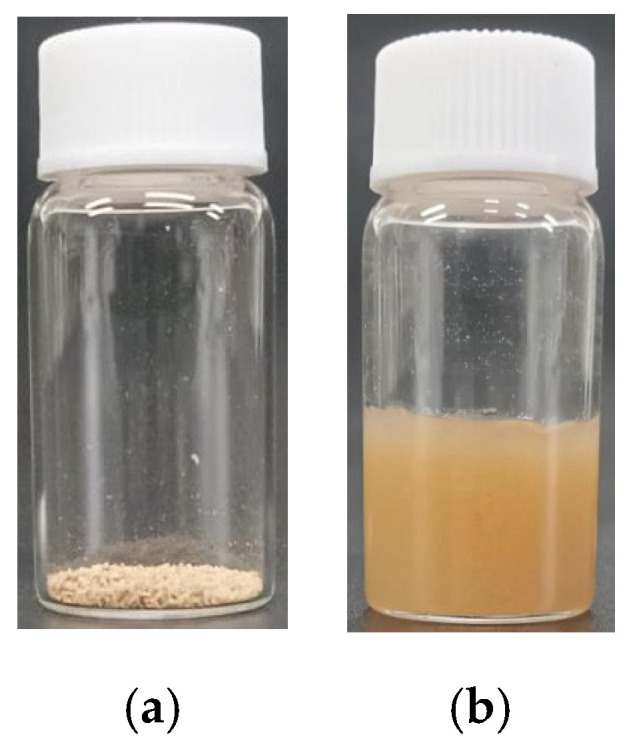
Visual representation of SAP (Entry 3) in glass vial (**a**) before and (**b**) after water absorption.

During the free swelling process, SAPs absorb half-bound water, bound water, and free water in the SAP structures. Among which free water is the most mobile and readily removed under centrifugal forces. During free swelling capacity measurements, water is trapped between interstitial spaces among SAP particles, and upon the application of centrifugal forces, the loosely bound water in the SAP is isolated and quantified. Therefore, CWR served as an effective indicator of the ability of SAP network to retain bound water after being subjected to centrifugation. Higher values of CWR correspond to enhanced water retention after centrifugation [46]. As shown in Figure 6, the CWR value of all the sample ranged from 6.8 to 15.8 g/g. The highest CWR value (15.8 g/g) was exhibited by SAP (Entry 3), indicating that it had retained much of the bound water in porous network structure by hydrogen bonding and electrostatic interactions and possessed low degree of crosslinking. On the other hand, SAP (Entry 11) showed low CWR (10.5 g/g) indicating that it possessed a higher degree of crosslinking. Similarly, the lowest CWR value of SAP (Entry 7) (6.78 g/g) and SAP (Entry 12) (7.2 g/g) revealed that the crosslinking density restricted water absorption in the SAPs’ network structures. The visual representation of water absorption of SAP (Entry 3) is depicted in Figure 7.

### 2.5. Proposed Reaction Mechanism

**Figure 8:**
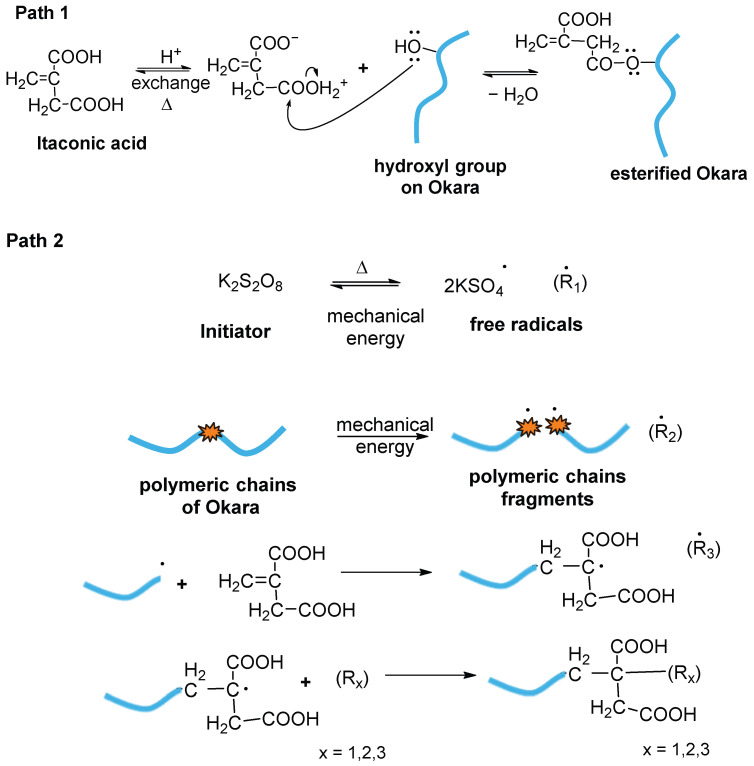
Proposed mechanisms for mechanochemical esterification of Okara with ItA.

In absence of the KPS initiator, the reaction occurred through the esterification process as described in Figure 8 (Path 1). In the presence of the KPS initiator, the reaction proceeded, along with Path 1, through a radical-initiated process as described in Figure 8 (Path 2). Thermal energy generated KPS radicals [38], and mechanical forces generated mechano-radicals [47,48]. The unpaired electrons on these radicals initiated a reaction by attacking the C=C bond of ItA, resulting in ItA being grafted onto Okara. The C=C double bond of ItA converted into C-C single bonds during free radical reactions, as evidenced by ATR-FTIR spectra that peaks at 1619 cm^−1^ due to C=C stretching and 898 cm^−1^ due to vinyl =CH_2_ bending disappearing completely. In the case of reactions using the MBA crosslinker, which also contains C=C double bonds, the incorporation of MBA into the SAP was indicated by the absence of C=C stretching vibrations in the ATR-FTIR spectra and an increase in the crystallinity of the SAP, as shown in Figure 2. Therefore, the reaction was believed to proceed through two possible pathways: one was the Fischer esterification reaction (Figure 8, Path 1) between ItA and -OH groups on Okara and the other one involved free radical mechanism, even in the absence of KPS. The mechanism of free radical generation and ItA grafting onto Okara is depicted in Figure 8, Path 2.

## 3. Materials and Methods

### 3.1. Materials

Ultra-fine 150 mesh size Okara powder was purchased from Yutec Co., Ltd. (Hokkaido, Sapporo, Japan). Itaconic acid (ItA, purity >99.0%) was purchased from Tokyo Chemical Industry (TCI, Tokyo, Japan). NaOH (purity 97.0%) and potassium peroxodisulfate (KPS, purity 98.0%) were purchased from Kanto Chemical Co., Inc., (Tokyo, Japan). *N*,*N′*-methylenebis(acrylamide) (MBA, ≥99%) was purchased from FUJIFILM Wako Pure Chemical Co., (Osaka, Japan). All the chemicals were used as received without further purification.

Microcompounder conical twin-screw extruder (Xplore MC 5, Xplore Instruments BV, Sittard, The Netherlands) was used to esterify Okara with ItA.

### 3.2. Mechanochemical Synthesis of SAPs

The method used for preparation of SAP (Entry 12) (see Table 2) will be discussed here as a representative example. In a typical procedure 1.78 g Okara, 2.86 g ItA, 1 wt% KPS (46.4 mg), and 0.5 wt% (23.2 mg) of MBA were mixed. They were introduced into the kneading barrel of microcompounder pre-heated at 120 °C. The reaction was carried out at 120 °C for 30 min. Afterwards, the crude solid product was recovered and grounded into powder using a mixer. Then, the product was dispersed into 100 mL distilled water before neutralization with 4% NaOH solution to pH 7 using a pH meter. After neutralization the SAP was precipitated with 50% acetone before filtration with filter paper and a Buchner funnel, followed by washing twice with water and acetone. Once purified, the SAP was dispersed in 50 mL distilled water and freezed. Then the SAP was freeze-dried for 89 h before measuring ATR-FTIR and undergoing further analysis. The reaction yield was calculated based on the mass of the final SAP obtained (*Y_Ok_* (%)) with respect to the initial mass of Okara, and was determined using Equation (1):

(1) $$Y_{Ok}(\%)=\frac{W_{SAP}}{W_{Okara}}\times 100$$

In the formula, *W_SAP_* is the weight of the dried, washed SAP, and *W_Okara_* is the initial weight of Okara powder, excluding the mass of KPS initiator and crosslinker MBA.

### 3.3. Characterization

#### 3.3.1. Measurement of ATR-FTIR

All Fourier-transform infrared (FTIR) spectra of Okara and its SAPs with ItA were recorded in attenuated total reflection (ATR) mode using a Thermo Fisher Scientific Nicolet iS10 (Thermo Fisher Scientific, Inc., Tokyo, Japan) spectrophotometer at a resolution of 4 cm^−1^ with an average of over 64 scans in the range of 400–4000 cm^−1^. The spectra were plotted after normalization using OriginPro 2026 SR1 software (OriginLab Corporation, Northampton, MA, USA).

#### 3.3.2. X-Ray Diffraction (XRD) Measurements

The X-ray diffraction analysis of Okara and its SAPs prepared by esterification with ItA were carried out using a D2 Phaser (2nd Gen) benchtop diffractometer (Bruker, Karlsruhe, Germany). It was equipped with Cu-Kα radiation (λ = 0.154 nm) and the measurements were made in the range of 2θ = 5 to 60°.

#### 3.3.3. Scanning Electron Microscopy (SEM) Measurements

Scanning electron microscopy (SEM) images of Okara and its SAPs with ItA were examined at an operating voltage of 10 kV on a JEOL JSM-6510LV scanning electron microscope (Akishima, Japan). The samples for SEM micrographs were prepared by mounting on aluminum stubs with conductive carbon tape, followed by sputter-coating of a thin layer of platinum using Magnetron sputtering equipment (MSP-20UM, Vacuum Device Inc., Mito-city, Ibaraki, Japan) under high vacuum to ensure electrical conductivity. The images were obtained under high vacuum at magnifications ×400 or ×500.

#### 3.3.4. Thermogravimetric Analysis (TGA)

Thermogravimetric analysis (TGA) of SAPs samples was performed using DTG-60AH/FC-60A/TA-60 (Shimadzu Co., Kyoto, Japan) in a temperature range of 25–600 °C, using the heating rate of 10 °C min^–1^ with constant flow of N_2_ gas at a rate of 50 mL min^–1^. For TGA measurements, 10 mg of each SAP sample was dried at 120 °C for 2 h before measuring the actual TGA curves. The onset thermal decomposition temperature was defined as a temperature at which ≥5% weight loss of SAPs was recorded and represented as T_onset_. The temperature at which maximum degradation of SAPs was recorded was labelled at T_max_. The amount of SAP remained after carbonization at 600 °C was reported as char residue as a percentage of SAP.

### 3.4. Measurement of Free Swelling Capacity

The free swelling capacity of the synthesized SAPs was measured in distilled water to evaluate their fluid absorption behaviour. The well-established Japanese Industrial Standard [JIS K 7223] tea bag method [49] was used to measure the free swelling capacity of SAP without any external pressure because it is more suitable and practical for time-dependent studies. The 10 × 20 cm tea bags were made by heat-sealing the nylon cloth (255 mesh, 57 μm opening). Then, carefully weighed 0.2 g SAP samples were placed in each tea bag and immersed in 1 L of distilled water to freely swell for 3 h. Following the absorption of water, the tea bags were hung in the air for 10 min to drain off excess water. The equilibrium swelling capacity (SC) was calculated in accordance with the following equation:

(2) $$SC\;(g/g)=\frac{W_{w}-W_{b}-W_{s}}{W_{s}}$$

where *W_s_*, *W_b_* and *W_w_* are the weights of initial dried SAP, the average weight of three pre-wetted tea bags, and the weight of swollen SAP in tea bags, respectively.

### 3.5. Measurement of Centrifugation Water Retention

Centrifugation water retention (CWR) is the amount of water retained by the SAP after dehydration by centrifugation for a specific time interval. It determines how much water is firmly bound by the ion–dipole and hydrogen bonding interactions of the SAPs, when subjected to centrifugal forces. The CWR of SAPs was determined using the tea bag method as reported by Huang et al. [46]. Nylon cloth was used to create tea bags of 10 × 10 cm size. After placing 0.20 g of SAP in each tea bag, they were heat sealed and immersed in distilled water. After swelling for 3 h, the tea bags were removed from water and centrifuged at 250 *g* for 3 min at 2 °C using Compact High Speed Refrigerated Centrifuge (Model 6000, KUBOTA Corporation Co., Ltd., Tokyo, Japan) to drain excess water. The amount of water retained by the SAP was measured and CWR was calculated by using Equation (3):

(3) $$CWR\;(g/g)=\frac{W_{c}-W_{tb}-W_{s}}{W_{s}}$$

where *W_s_*, *W_tb_* and *W_c_* are the weights of initial dried SAP, the average weight of three blank pre-wetted tea bags, and the weight of swollen SAP in a tea bag after centrifugation, respectively.

## 4. Conclusions

In summary, we have successfully grafted ItA onto the Okara by a mechanochemical solventless esterification process and prepared SAPs. The grafting process proceeded via Fischer esterification and free radical mechanisms. We found that the reaction can proceed smoothly without the KPS initiator, and similarly the presence of MBA crosslinker can be relinquished. For example, the SAP (Entry 5) prepared from 1.4 g/3.37 g ratio of Okara/ItA without the use of KPS and MBA exhibited modest water absorption of 20.8 g/g. This argument was further augmented by the fact that the SAP (Entry 3), which was prepared without the use of KPS showed the best water absorption ability of 35.6 g/g. The CWR capacity showed the same trend as free water swelling behaviour while retaining roughly 60% water absorption as compared to free swelling capacity for most of the SAPs. In conclusion, we propose that the grafting of ItA onto natural polymers by replacing petroleum-based acrylic acid using mechanochemistry can be adapted to existing manufacturing processes for synthesis of SAPs where high water absorption is not required.

## Figures and Tables

**Table 1:** Thermogravimetric data of Okara and its SAPs mechanochemically synthesized with ItA.

Sample	T_onset_ (°C)	T_max_ (°C)	Char Residue at 600 °C (%)
Okara	245	275, 338.68	25.27
Entry 1	238	317.25	29.67
Entry 2	228	304.09	30.20
Entry 3	244	331.77	30.57
Entry 4	242	319.45	38.52
Entry 5	248	276, 314.19	28.99
Entry 6	244	324.95	30.85
Entry 7	246	280, 327.05	35.23
Entry 8	240	324.74	39.30
Entry 9	249	280, 316.84	37.14
Entry 10	249	324.74	29.79
Entry 11	237	286, 314.60	39.13
Entry 12	238	312.89	34.03

**Table 2:** The weights of Okara and ItA used for preparation of SAPs along with wt% of free radical initiator (KPS) and crosslinker (MBA), and the mass yield based on Okara.

SAP Sample	Okara (g)	ItA (g)	KPS (wt % Total)	MBA (wt % Total)	$Y_{Ok}$ **(%)**
Entry 1	1.00	3.50	-	-	61.09
Entry 2	1.00	3.50	0.5	-	43.73
Entry 3	1.00	3.50	-	0.5	39.37
Entry 4	1.00	3.50	0.5	0.5	65.58
Entry 5	1.40	3.37	-	-	56.85
Entry 6	1.40	3.37	2.0	-	64.21
Entry 7	1.40	3.37	-	0.5	77.31
Entry 8	1.40	3.37	2.0	0.5	86.41
Entry 9	1.78	2.86	-	-	81.44
Entry 10	1.78	2.86	1.0	-	69.34
Entry 11	1.78	2.86	-	0.5	56.84
Entry 12	1.78	2.86	1.0	0.5	73.80

## Data Availability

The original contributions presented in this study are included in the article. Further inquiries can be directed to the corresponding author.

## Supplementary Materials

The following supporting information can be downloaded at: https://www.mdpi.com/article/10.3390/molecules31111830/s1, Figure S1: ATR mode FTIR of spectra of SAPs prepared with the use of KPS radical initiator; Figure S2: ATR mode FTIR of spectra of SAPs prepared without the use of KPS radical initiator; Figure S3: X-ray diffractograms of SAPs (Entries 4 to 12) prepared with and/or without KPS initiator and MBA crosslinker; Figure S4: Thermogravimetric analysis (TGA) graphs of SAPs (Entries 5 to 12) synthesized by mechanochemical reaction of Okara with ItA with and/or without KPS and MBA; Figure S5: DTG curves of Okara and Its SAPs prepared with ItA.
