# Supplementary material for: Mechanochemical Preparation of Superabsorbent Materials from Okara and Itaconic Acid
Source: Molecules. 2026 May 26;31(11):1830. doi: 10.3390/molecules31111830 (PMC13257813; doi:10.3390/molecules31111830)
Supplement: Supplementary file 1 [file molecules-31-01830-s001.zip › molecules-4307059-supplementary.pdf]

Supplementary Information

Mechanochemical Preparation of Superabsorbent Materials from Okara and Itaconic Acid

Abdul Hafeez [1,*], Gyanendra Sharma [2], Romain Milotskyi [2], Hao Wang [1], Akihiro Shinku [1], Naoki Wada [2] and Kenji Takahashi [2,*]

[1] Division of Biological Science and Technology, Graduate School of Natural Science and Technology, Kanazawa University, Kakuma-Machi, Kanazawa 920-1192, Ishikawa, Japan; h-wang@se.kanazawa-u.ac.jp (H.W.); shinaki0925sitwa@stu.kanazawa-u.ac.jp (A.S.)

[2] Faculty of Biological Science and Technology, Institute of Science and Engineering, Kanazawa University, Kakuma-Machi, Kanazawa 920-1192, Ishikawa, Japan; sharmag-19@se.kanazawa-u.ac.jp (G.S.); romain-mi@se.kanazawa-u.ac.jp (R.M.); naoki-wada@se.kanazawa-u.ac.jp (N.W.)

* Correspondence: hafeez163@stu.kanazawa-u.ac.jp (A.H.); ktkenji@staff.kanazawa-u.ac.jp (K.T.); Tel.: +81-90-1395-4507 (A.H.); +81-76-234-4828 (K.T.)

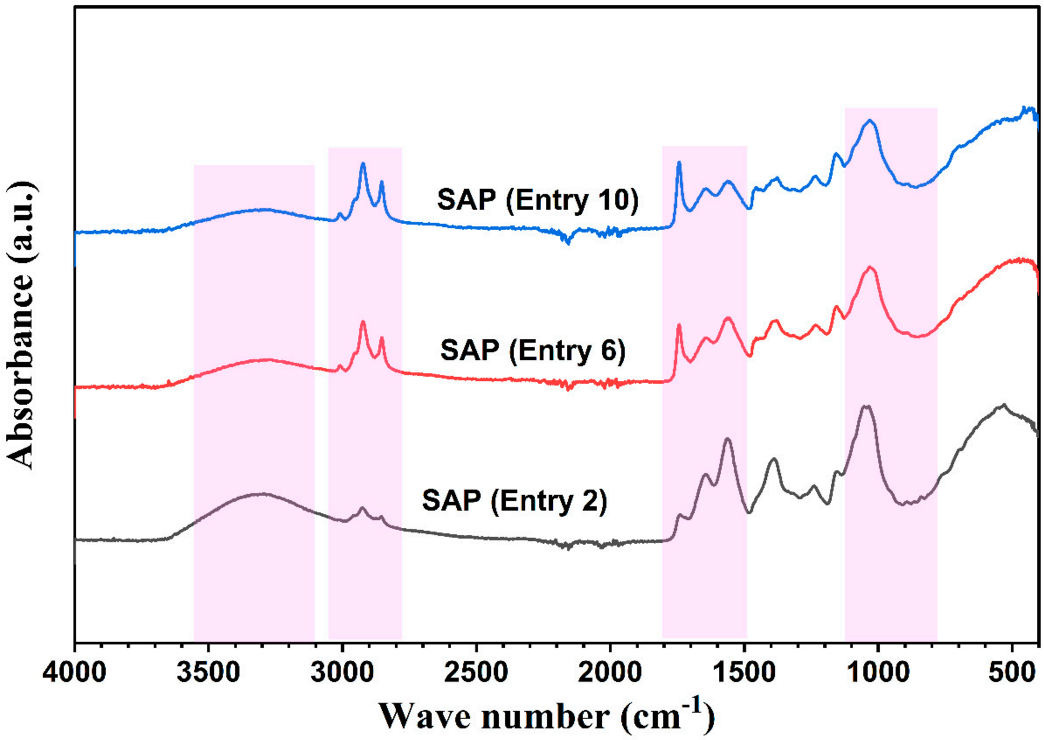

Figure S1. ATR mode FTIR of spectra of SAPs prepared with the use of KPS radical initiator.

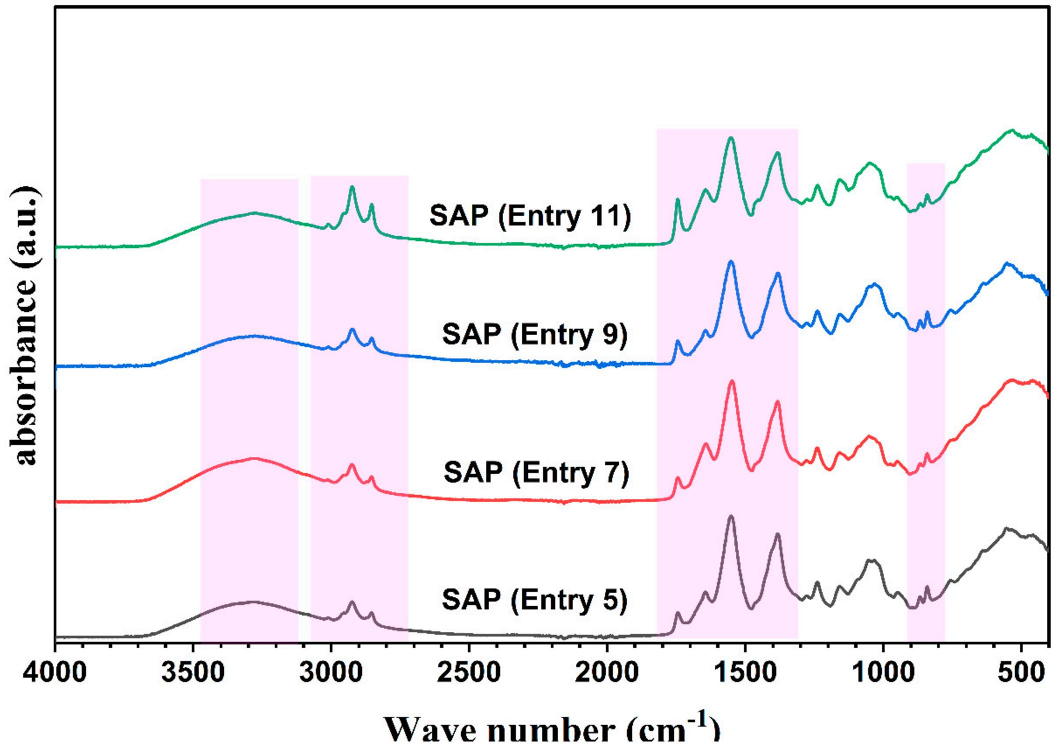

Figure S2. ATR mode FTIR of spectra of SAPs prepared without the use of KPS radical initiator.

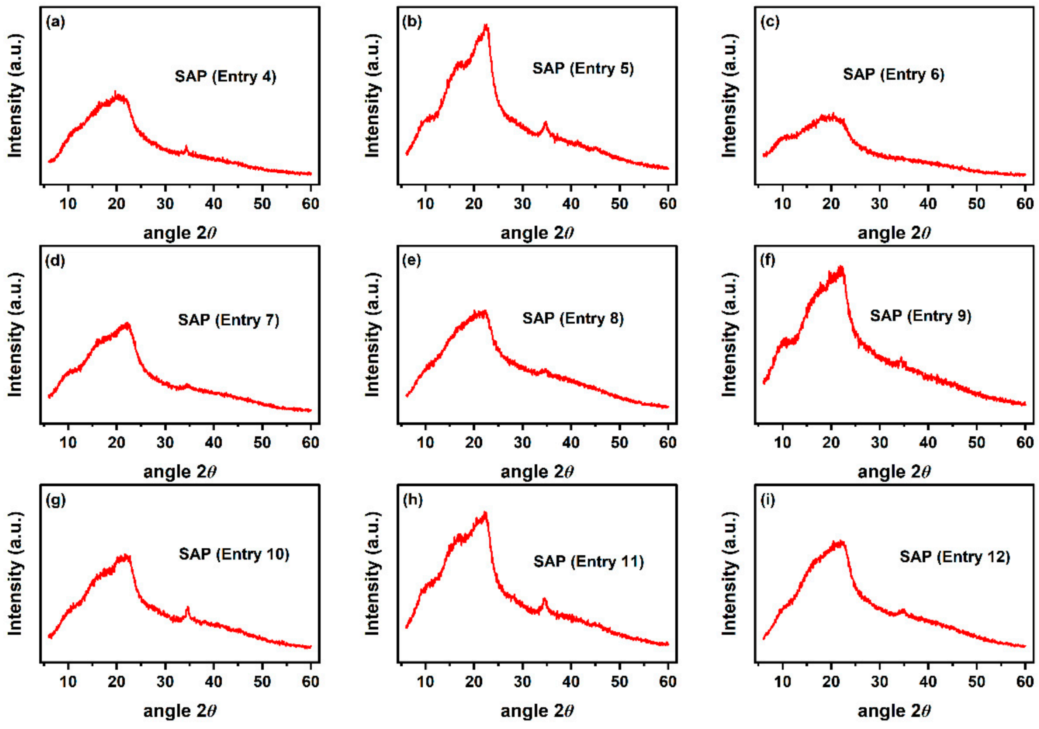

Figure S3. X-ray diffractograms of SAPs (Entries 4 to 12) prepared with and/or without KPS initiator and MBA cross-linker.

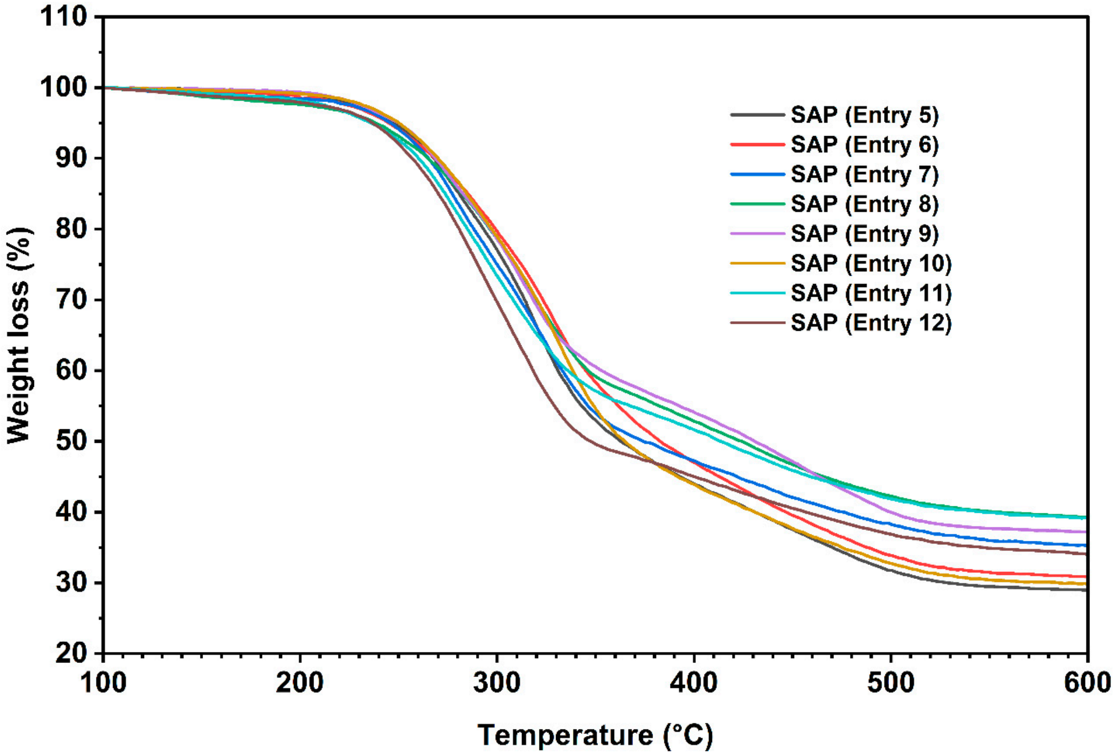

Figure S4. Thermogravimetric analysis (TGA) graphs of SAPs (Entries 5 to 12) synthesized by mechanochemical reaction of Okara with ItA with and/or without KPS and MBA.

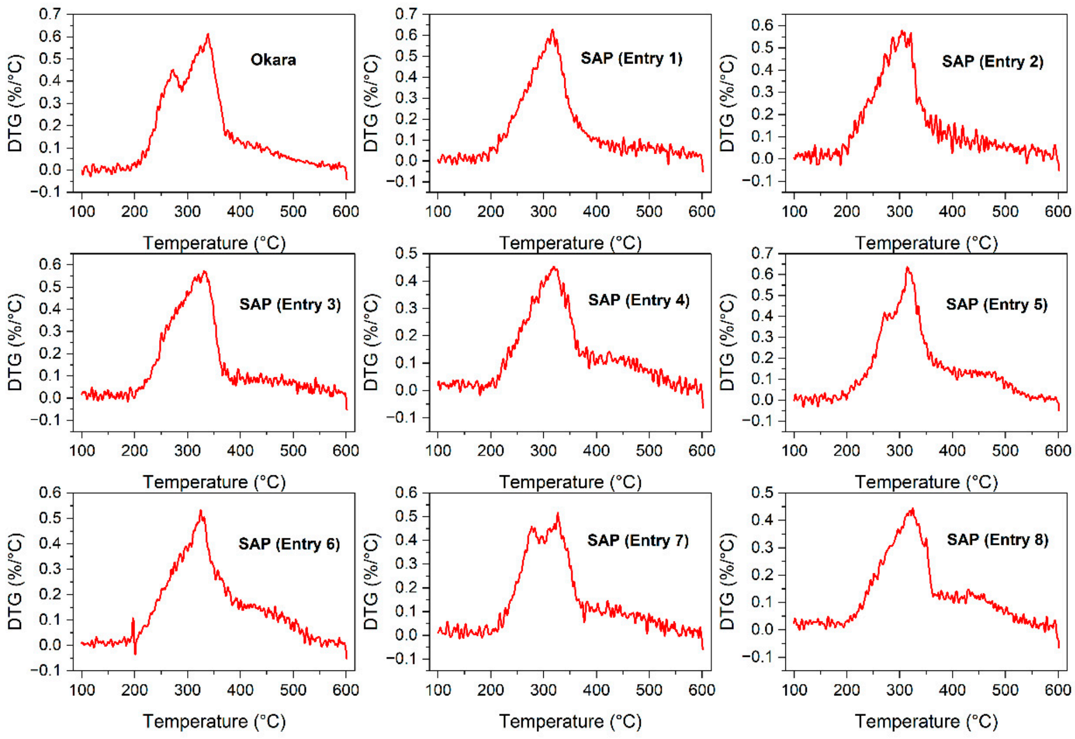

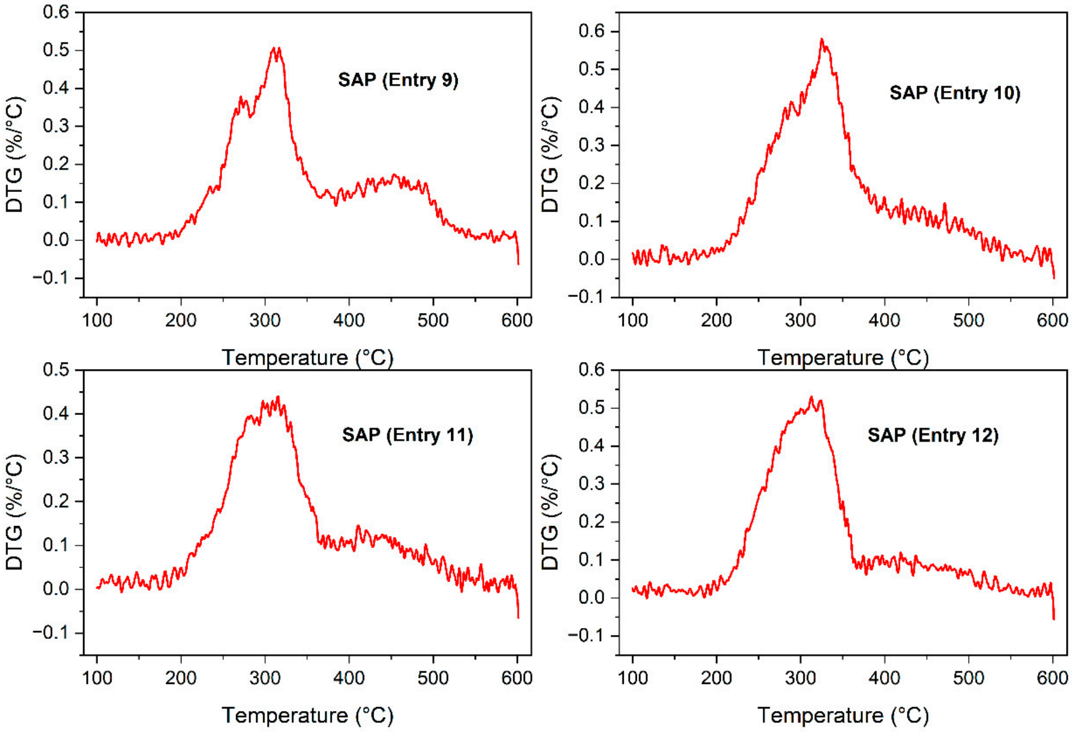

Figure S5. DTG curves of Okara and Its SAPs prepared with ItA.
